# Perpetual observational study of the clinical and microbiological epidemiology of ventilator-associated pneumonia in Europe

**DOI:** 10.1186/s13054-025-05753-5

**Published:** 2026-03-12

**Authors:** Holly Jackson, Ana Catalina Hernandez Padilla, Lisanne E. M. Vintcent, Aleksandra Barac, Olaf Cremer, Thomas Daix, Jan J. De Waele, Lorena Forcelledo, Olivier Barraud, Marc J. M. Bonten, Stephan Harbarth, Bruno Francois, C. H. van Werkhoven, Marlieke E. A. de Kraker, the ECRAID-Base Consortium, Holly Jackson, Ana Catalina Hernandez Padilla, Lisanne E. M. Vintcent, Olivier Barraud, Marc J. M. Bonten, Stephan Harbarth, Bruno Francois, C. H. van Werkhoven, Marlieke E. A. de Kraker

**Affiliations:** 1 Infection Control Program, Geneva University Hospitals and Faculty of Medicine, World Health Organization Collaborating Center, Geneva, Switzerland; 2 Inserm CIC 1435, CHU Limoges, Limoges, France; 3 Inserm UMR 1092, Université de Limoges, Limoges, France; 4 Service de Réanimation Polyvalente, CHU Limoges, Limoges, France; 5 Julius Center for Health Sciences and Primary Care, University Medical Center Utrecht, Utrecht University, Utrecht, The Netherlands; 6 University Clinical Center of Serbia, Belgrade, Serbia; 7 Department of Intensive Care Medicine, University Medical Center Utrecht, Utrecht University, Utrecht, The Netherlands; 8 Department of Intensive Care Medicine, Ghent University Hospital, Gent, Belgium; 9 Department of Internal Medicine and Pediatrics, Faculty of Medicine and Health Sciences, Ghent University, Ghent, Belgium; 10 Hospital Universitario Central de Asturias (HUCA), Oviedo, Spain; 11 Group of Translational Microbiology, Health Research Institute of Principado de Asturias (ISPA), Oviedo , Spain; 12 Service de Bactériologie-Virologie-Hygiène, CHU Limoges, Limoges, France; 13 European Clinical Research Alliance on Infectious Diseases (Ecraid), Utrecht, The Netherlands

**Keywords:** Ventilator-associated pneumonia, Epidemiology, Microbiology, Intensive care unit, Healthcare-acquired infection, Outcomes, Prevention measures

## Abstract

**Background:**

The clinical and microbiological epidemiology of ventilator-associated pneumonia (VAP) is not well studied in intensive care units (ICUs) European wide. The European Clinical Research Alliance on Infectious Diseases (Ecraid), a warm-base clinical research network investigating infectious diseases, aimed to track the implementation of VAP prevention strategies and quantify the incidence, aetiology, and clinical outcome of VAP, across several European countries.

**Methods:**

Overall, 25 ICUs from 11 European countries participating in Ecraid’s perpetual observational study prospectively enrolled adult patients with an expected length of invasive mechanical ventilation (IMV) of at least 48 h, between August 2022 and September 2024. VAP was defined according to the US Food and Drug Administration guidelines. Patients were followed until ICU discharge or 28 days after VAP diagnosis. Routine clinical and microbiological data were prospectively collected. Mortality was calculated using cumulative incidence functions.

**Results:**

Of the 3,446 patients at-risk of VAP, 590 developed VAP (cumulative incidence: 17.1%, 95% CI 15.9%-18.4% and incidence rate per 1000 ventilator days: 18.6, 95% CI 17.1–20.1). Importantly, VAP cumulative incidence varied widely between countries recruiting at least 100 patients (range: 7.6% (Croatia)-29.6% (Romania)). Microbiological documentation was available for 359 (60.8%) VAP patients, predominantly showing *Staphylococcus aureus* (26.2%), *Haemophilus influenzae* (16.2%), and *Pseudomonas aeruginosa* (15.0%). Methicillin resistance was confirmed in 14 (18.2%) of 77 VAP cases due to *S. aureus*. Ceftazidime and carbapenem resistance for *P. aeruginosa* was reported in 10/46 (21.7%) and 8/47 (17.0%) cases, respectively. Cumulative incidence of ICU mortality was 34.2% (95% CI 30.4%-38.0%) among VAP patients versus 29.3% (95% CI 27.6%-30.9%) in non-VAP patients. The overall median IMV duration until first extubation was 17 days in VAP patients (including ventilation before and after diagnosis) versus 7 days for non-VAP patients. The most widely implemented VAP prevention measure was head-of-bed elevation (3207 patients, 93.1%); only 4 patients (0.1%) did not have any prevention measures implemented.

**Conclusion:**

In European ICUs, there is a considerable and heterogeneous incidence of VAP, with methicillin susceptible *S. aureus* most frequently identified as a causative pathogen. VAP is associated with poor clinical prognosis, highlighting the need for better VAP prevention and management strategies.

**Supplementary Information:**

The online version contains supplementary material available at 10.1186/s13054-025-05753-5.

## Background

Many critically ill patients who are admitted to intensive care units (ICUs) require invasive mechanical ventilation (IMV). Although IMV is a life-saving intervention, it is also associated with complications, including ventilator-associated pneumonia (VAP) [1], which is the most common hospital acquired infection in ICUs [2]. The reported cumulative incidence of VAP ranges between 4 and 40% [3, 4], dependent on ICU settings and applied diagnostic criteria. VAP poses a significant burden to patients admitted to the ICU, the estimated attributable mortality ranges between 0 and 27% [5–9]. Furthermore, VAP is associated with increased length of stay, antibiotic prescribing and costs [10]. Several preventive strategies have been suggested as single interventions or combined in prevention bundles [11]. Despite their widespread implementation, VAP incidence remains high [12].

There is no harmonised, objective definition of VAP, diagnosis often relies on clinical signs and symptoms, typically in combination with presence of new infiltration on a chest CT or X-ray [13, 14]. This hinders comparison of results between studies or aggregation of data about VAP incidence and/or aetiology to inform clinical trial designs. The Perpetual Observational Study in Ventilator-Associated Pneumonia (POS-VAP) is part of the European Clinical Research Alliance on Infectious Diseases (Ecraid), a warm-base clinical research network. Patients are prospectively enrolled to collect routine clinical and microbiological data related to VAP, and this perpetual study can serve as a backbone to efficiently embed preventive, diagnostic, or therapeutic studies to improve management of patients under IMV [15, 16].

We describe the clinical and microbiological epidemiology of VAP, including incidences, clinical characteristics, standard-of-care strategies for VAP prevention and treatment, microbiological results, and outcomes, among ICU patients across eleven European countries.

## Methods

### Study design

POS-VAP is a multicentre, prospective, observational study, including adult patients at risk of VAP (ClinicalTrials.gov ID: NCT05719259). The aim of POS-VAP is to build a sustainable European clinical research network of ICUs that can serve as a platform to facilitate observational and randomised VAP research activities [16]. This analysis describes implementation of standard of care strategies for VAP prevention and treatment, and quantifies the incidence, aetiology, and clinical outcome of VAP across several European countries, for patients recruited between 4th August 2022 and 12th September 2024.

### Study sites

Of 105 sites invited to submit feasibility data, 60 sites were selected based on availability of at least 10 ICU beds with IMV, GCP certified investigator(s) and personnel capacity, with a focus on tertiary care university hospitals. Sites opened at varying times throughout the study timeline (Supplementary Table 1). As of 12/09/2024, 25 ICUs (88% mixed ICUs, 76% from university hospitals) were recruiting patients across 11 European countries: Albania (*n* = 1 ICU), Belgium (*n* = 4), Czech Republic (*n* = 2), Spain (*n* = 3), France (*n* = 6), Greece (*n* = 1), Croatia (*n* = 2), Italy (*n* = 1), the Netherlands (*n* = 2), Romania (*n* = 2), and Serbia (*n* = 1).

### Patient inclusion/exclusion criteria

ICU patients > 18 years, with an expected or documented length of IMV of ≥ 48 h were eligible. Patients were excluded if death was deemed imminent or inevitable at hospital admission (i.e. within 48 h as judged by the attending physician) and/or the patient, substitute decision maker, or attending physician were not committed to full treatment.

### Analysis populations and endpoints

We defined four analysis populations for different endpoints: the at-risk population, VAP population, microbiologically evaluable (MBE) VAP population and the antibiotic treated (ABT) VAP population (Table 1, definitions in Supplementary Table 2).

**Table 1:** Analysis populations and their study endpoints

Analysis population	Endpoints per population
The **at-risk population** included all enrolled patients (with or without VAP) that had a documented duration of IMV of at least 48 h Patients stay at-risk of VAP up to 48 h after they are removed from the ventilator [17]	First episode of VAP, all-cause mortality in the ICU, overall length of ICU stay, and overall length of IMV
The **VAP population** was defined as all patients diagnosed with VAP according to the US Food and Drug Administration (FDA) criteria (the presence of at least one clinical respiratory feature and systemic sign, and a chest image suggestive of bacterial pneumonia as determined by the local clinical team [18]), during their first ventilation episode. See definitions in Supplementary Table 2	28 day all-cause mortality after VAP diagnosis, length of ICU stay before and after VAP diagnosis, length of IMV before and after VAP diagnosis, number of IMV-free days within the 28 days after VAP diagnosis, and clinical cure (Supplementary Table 2)
The **MBE VAP population** included all VAP patients who had at least one microbiological sample taken within +/−2 days from their VAP diagnosis date, in which a relevant VAP pathogen was identified (i.e. they had a microbiologically documented infection)	Frequency of VAP pathogens identified, their resistance proportions, and microbiological cure (Supplementary Table 2)
The **ABT VAP population** incorporated the VAP patients who also had a physician diagnosis of VAP within +/−1 day of their FDA VAP diagnosis date, and who started an antibiotic treatment for this indication within +/−1 day of their physician diagnosis of VAP	Antibiotic administration by class. The antibiotics included in each antibiotic class are displayed in Supplementary Table 3

### Patient data collection

Data were collected prospectively via an electronic Case Report Form. Baseline data included demographics, past medical history, reason for ICU admission, comorbidities, date of intubation, hospital admission and ICU admission, severity scores (Acute Physiology and Chronic Health Evaluation [19], Sequential Organ Failure Assessment [20], Charlson comorbidity index [21], and application of VAP prevention strategies. Screening for VAP occurrence was performed by treating physicians based on their standard of care. Researchers were requested to enter data relative to VAP screening whenever a chest image was prescribed for VAP suspicion. The US Food and Drug Association (FDA) criteria were assessed when a chest image showed an infiltrate suggestive of VAP. Upon meeting the US FDA VAP criteria, additional collected data included: microbiological tests within +/−2 days of meeting VAP criteria; clinical and microbiological cure between days 7–10 after VAP; ventilation episodes during their ICU stay up to day 28 after VAP. Patients were followed until ICU discharge, additionally, for VAP patients, vital status was assessed at day 28. Data collected at ICU discharge included: vital status, physician VAP diagnosis and VAP related antibiotics. Data quality was improved through built-in data checks, remote monitoring, and external data verification with feedback rounds with participating sites.

### Statistical methods

Descriptive statistics were used to summarise all endpoints, including median and inter-quartile ranges (IQR) for non-normally distributed continuous variables and frequencies and percentages for categorical variables.

Non-parametric cumulative incidence functions (CIFs) with 95% confidence intervals (CIs) were plotted and used to derive: VAP incidence (including extubation, ICU death and ICU discharge as competing events), ICU mortality (including ICU discharge as a competing event) for VAP and non-VAP patients, and 28-day all-cause mortality (labelling those patients with missing data as censored at the date they were discharged alive) for VAP patients.

Stacked probability plots were utilised to display the probability of a patient being in each state (VAP and successful extubation were intermediate states, ICU discharge and ICU death were absorbing states) of our multistate extended illness-death model (Supplementary Fig. 1) [22].

As this is a descriptive analysis, missing data were reported but not imputed. For CIFs, patients were censored at the relevant date.

All analyses were performed using R Statistical Software (version 4.4.2) [23].

## Results

### Inclusion and baseline characteristics

**Fig. 1:**
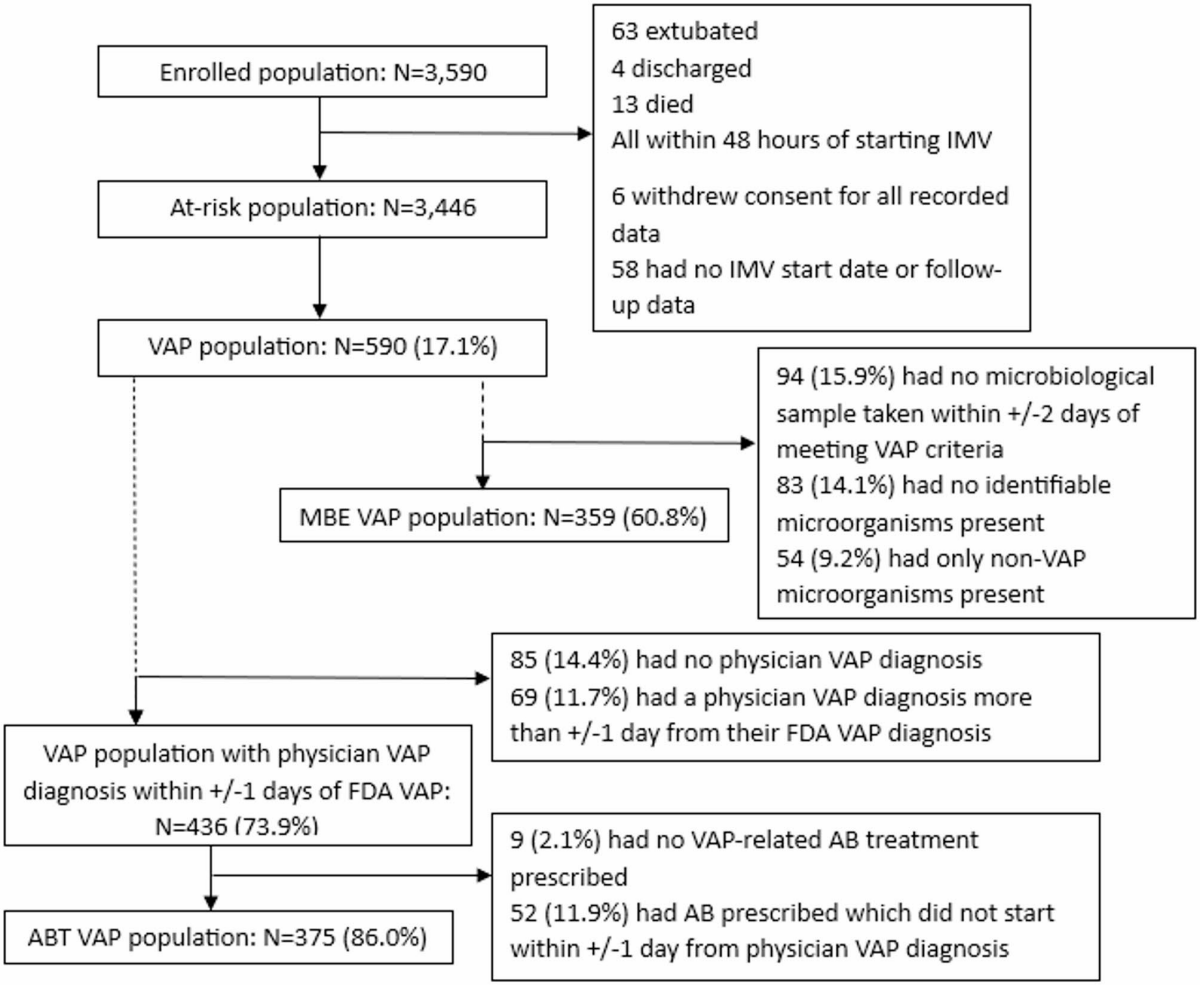
Inclusion flowchart and size of analysis populations. IMV, invasive mechanical ventilation; VAP, ventilator-associated pneumonia; FDA, Food and Drug Administration; MBE, microbiologically evaluable; AB, antibiotic; ABT, antibiotic treated; Solid line indicates patients are split between subpopulations; Dotted lines indicate patients can be in multiple subpopulations

Over 25 months, 3,590 patients were enrolled within POS-VAP, of which, 3,446 patients were ‘at-risk’ (Fig. 1). The top recruiting countries were France (1210, 35.1%), Serbia (567, 16.5%) and the Netherlands (353, 10.2%) (Supplementary Table 1).

Overall, 2,251 (65.3%) participants were male, and the median age was 64 years old (IQR 52–72). The most frequent reason for ICU admission was acute respiratory failure (*n* = 580, 16.8%). The most common comorbidities were uncomplicated diabetes mellitus (*n* = 549, 15.9%) and congestive heart failure (*n* = 464, 13.5%). VAP patients were younger and more frequently male (Table 2). VAP patients were more often admitted to the ICU due to stroke and trauma, while non-VAP patients were more frequently admitted due to sepsis or as a planned postoperative ICU admission.

**Table 2:** Baseline characteristics for the at-risk population (*N* = 3,446) stratified by VAP diagnosis

Category	VAP (*N* = 590)	Non-VAP (*N* = 2,856)	Total (*N* = 3,446)	SMD
Demographics				
Age	61 (49–70)	64 (53–73)	64 (52–72)	0.20
Sex				
Male	418 (70.8)	1,833 (64.2)	2,251 (65.3)	0.14
*Comorbidities at ICU admission*				
Diabetes mellitus				0.06
Yes, end-organ damage Yes, uncomplicated None/diet controlled	17 (2.9) 84 (14.2) 489 (82.9)	76 (2.7) 465 (16.3) 2,315 (81.1)	93 (2.7) 549 (15.9) 2,804 (81.4)	
Congestive heart failure	72 (12.2)	392 (13.7)	464 (13.5)	0.05
Solid tumour				0.08
Yes, metastatic Yes, localised No	8 (1.4) 52 (8.8) 530 (89.8)	66 (2.3) 283 (9.9) 2,507 (87.8)	74 (2.1) 335 (9.7) 3,037 (88.1)	
COPD	66 (11.2)	293 (10.3)	359 (10.4)	−0.03
Cerebrovascular accident (CVA) or Transient ischemic attack (TIA)	40 (6.8)	250 (8.8)	290 (8.4)	0.07
Chronic kidney disease	37 (6.3)	217 (7.6)	254 (7.4)	0.05
Peripheral vascular disease	47 (8.0)	192 (6.7)	239 (6.9)	−0.05
Myocardial infarction	44 (7.5)	182 (6.4)	226 (6.6)	−0.04
Liver disease				0.09
Moderate to Severe Mild None	13 (2.2) 13 (2.2) 564 (95.6)	108 (3.8) 59 (2.1) 2,689 (94.2)	121 (3.5) 72 (2.1) 3,253 (94.4)	
Connective tissue disease	16 (2.7)	74 (2.6)	90 (2.6)	−0.01
Dementia	18 (3.1)	63 (2.2)	81 (2.4)	−0.05
Peptic ulcer disease	10 (1.7)	66 (2.3)	76 (2.2)	0.04
Hemiplegia	12 (2.0)	61 (2.1)	73 (2.1)	0.01
Lymphoma	11 (1.9)	54 (1.9)	65 (1.9)	0.00
Leukemia	5 (0.8)	56 (2.0)	61 (1.8)	0.09
AIDS	3 (0.5)	9 (0.3)	12 (0.3)	−0.03
*Reason for ICU admission*				0.28
Acute respiratory failure (hypoxemic/hypercapnic) Stroke (ischemic, hemorrhagic, subarachnoid hemorrhage) Trauma (any type of blunt or penetrating trauma) Traumatic brain injury Sepsis/Septic shock (excluding pulmonary origin) Altered mental status, coma Post-cardiac arrest Planned postoperative ICU admission (excluding cardiovascular surgery) Community acquired pneumonia (Any cause, except SARS-CoV-2) Abdominal disorder (including pancreatitis and mesenteric ischemia) Cardiovascular surgery (including aortic aneurysm) Cardiac decompensation or cardiogenic shock (excludes cardiac arrest) Haemorrhagic shock or severe GI bleeding Other CNS disorder (infection/solid lesion/hydrocephalus) Neurosurgery Acute myocardial infarction (STEMI or NSTEMI) Status epilepticus or other frequent/refractory seizures COVID-19 Shock (undifferentiated, other aetiology) Hospital-acquired pneumonia Burn Other	97 (16.4) 73 (12.4) 66 (11.2) 37 (9.2) 34 (5.8) 35 (5.9) 35 (5.9) 18 (3.1) 23 (3.9) 16 (2.7) 22 (3.7) 16 (2.7) 6 (1.0) 10 (1.7) 12 (2.0) 13 (2.2) 6 (1.0) 11 (1.9) 4 (0.7) 4 (0.7) 3 (0.5) 49 (8.3)	483 (16.9) 301 (10.5) 237 (8.3) 264 (9.2) 251 (8.8) 174 (6.1) 163 (5.7) 143 (5.0) 87 (3.0) 83 (2.9) 73 (2.6) 68 (2.4) 63 (2.2) 49 (1.7) 42 (1.5) 38 (1.3) 43 (1.5) 36 (1.3) 30 (1.1) 19 (0.7) 10 (0.4) 199 (7.0)	580 (16.8) 374 (10.9) 303 (8.8) 301 (8.7) 285 (8.3) 209 (6.1) 198 (5.7) 161 (4.7) 110 (3.2) 99 (2.9) 95 (2.8) 84 (2.4) 69 (2.0) 59 (1.7) 54 (1.6) 51 (1.5) 49 (1.4) 47 (1.4) 34 (1.0) 23 (0.7) 13 (0.4) 248 (7.2)	
*Illness severity scores*				
APACHE-II score at intubation				
Median (IQR) Missing	20 (16–24) 9	21 (16–26) 44	21 (16–26) 53	0.09
SOFA score at intubation				
Median (IQR) Missing	8 (6–10) 15	8 (6–10) 134	8 (6–10) 149	−0.01
Charlson comorbidity index at ICU admission				
Median (IQR) Missing	3 (1–5) 0	3 (1–5) 3	3 (1–5) 3	0.17
*VAP prevention measure at intubation (patients)*				
Head-of-bed elevation Daily oral care and chlorhexidine Peptic ulcer disease prophylaxis Daily “sedation vacation” All the above None of the above	543 (92.0) 412 (69.8) 377 (63.9) 329 (55.8) 204 (34.6) 0 (0.0)	2,664 (93.3) 1,889 (66.1) 1,681 (58.9) 1,707 (59.8) 1,042 (36.5) 4 (0.1)	3,207 (93.1) 2,301 (66.8) 2,058 (59.7) 2,036 (59.1) 1,246 (36.2) 4 (0.1)	0.05 −0.08 −0.10 0.08 −0.03

Data are given as n (%) for categories and median (interquartile range [IQR]) for continuous variables

VAP, ventilator-associated pneumonia; ICU, intensive care unit; SARS-CoV, severe acute respiratory syndrome coronavirus; GI, gastrointestinal; CNS, central nervous system; STEMI, ST elevated myocardial infarction; NSTEMI, non-ST elevated myocardial infarction; COVID, Coronavirus disease; APACHE, acute physiology and chronic health evaluation; SOFA, sequential organ failure assessment; SMD, standardised mean difference, was used to assess similarity between VAP and non-VAP patients. The SMD was calculated as the absolute value in the difference in means of a covariate across the two populations, divided by the pooled standard deviation [24]

### VAP preventive measures

The VAP prevention measures utilised, varied by ICU. All 25 ICUs implemented head-of-bed elevation in at least one patient, the median implementation across ICUs was 99.0% of patients (range: 11.1%−100% of patients within a single ICU) and all ICUs implemented daily ‘sedation vacation’ in at least one patient (median: 70.8%, range: 1.8%−100% of patients within a single ICU). Three ICUs did not implement daily oral care in any of their patients (median implementation: 97.0%, range: 0%−100% of patients per ICU) and two ICUs did not implement peptic ulcer disease prophylaxis in any of their patients (median implementation: 91.6%, range: 0%−100% of patients per ICU).

### VAP incidence

**Fig. 2:**
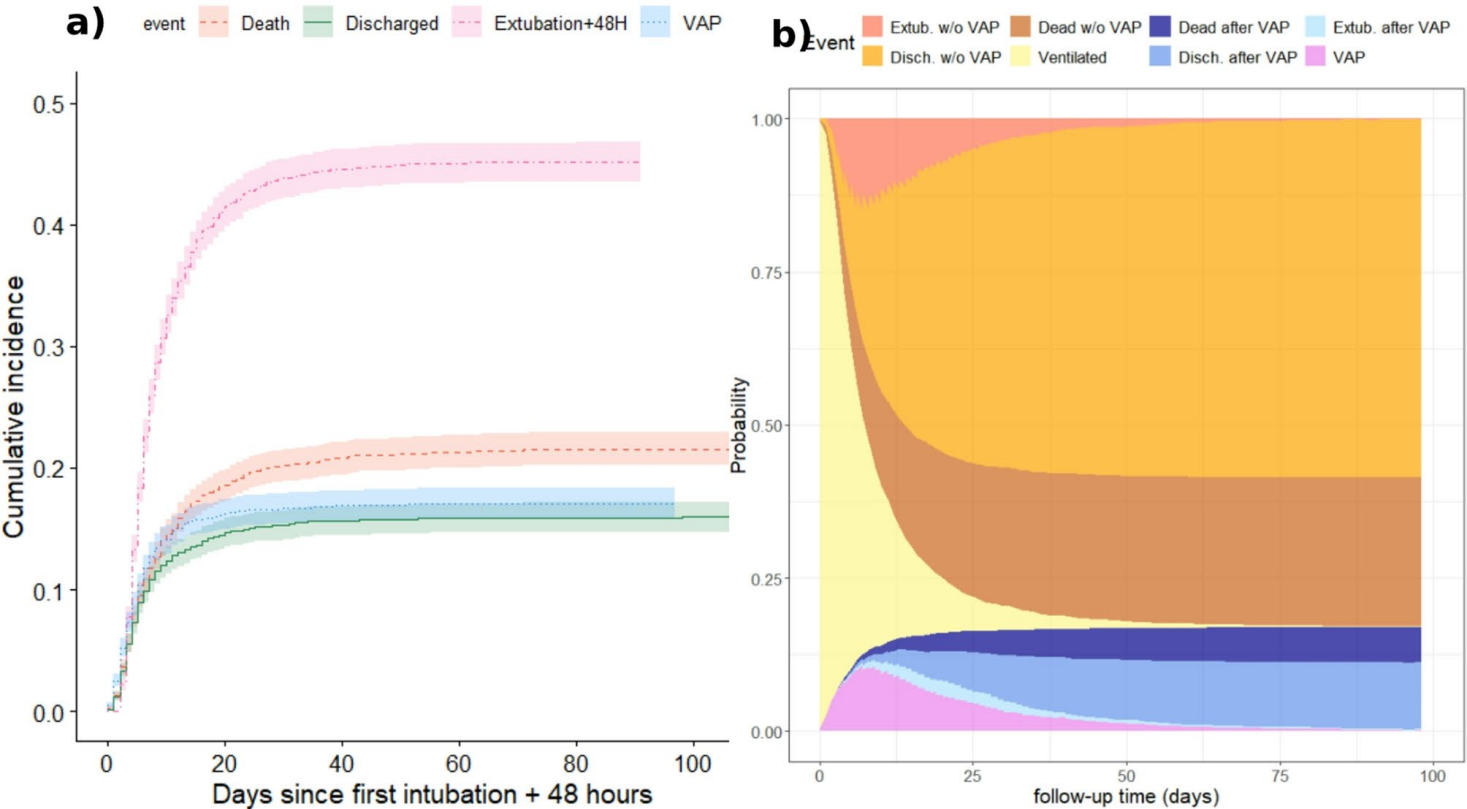
(**a**) Cumulative incidence of first ICU event: VAP, ICU death, ICU discharge, or successful extubation with 95% confidence intervals. (**b**) Stacked probability plot showing the probability that a patient is within each state over their length of follow-up [22]. At-risk population (*N* = 3,446)

Of 3,446 participants at risk of VAP, 590 (17.1%) met US FDA VAP criteria during their first episode of IMV. There were 185 (31.4%) patients with early-onset VAP (diagnosed $\:\le\:$4 days from intubation) and 405 (68.6%) patients with late-onset VAP (diagnosed >4 days after intubation) [25]. The cumulative incidence of VAP, considering competing events, was 17.1% (95% CI 15.9%−18.4%) (Fig. 2a) and the incidence of VAP per 1000 ventilator days was 18.6 (95% CI 17.1–20.1), both of which varied by country (Supplementary Fig. 2). Restricting our analysis to countries which recruited at least 100 patients, the cumulative incidence of VAP ranged between 7.6% (Croatia) and 29.6% (Romania) and the incidence of VAP per 1000 ventilator days ranged from 10.6 in Serbia to 30.3 in Romania. For patients at risk of VAP, there was a sharp increase in the probability of VAP and extubation without VAP in the first 5 days, followed by a more slowly increasing probability of ICU discharge or ICU death for patients with/without VAP (Fig. 2b).

There was high concordance between the FDA VAP criteria (590 VAP cases) and the physician-based VAP diagnosis (610 VAP cases). Of the 590 VAP patients, 505 (85.6%) were also diagnosed by their attending physician. There were 105/610 patients (17.2%) who were diagnosed with VAP by their attending physician, but who did not fulfil the FDA VAP criteria.

### Microbiological results

Overall, 582 VAP relevant pathogens were identified from a total of 425 positive samples taken in 359/590 (60.8%) VAP patients, labelled as the MBE VAP population. Among VAP patients without a sample within 48 h, 35/94 (37.2%), had no physician VAP diagnosis within 1 day of FDA VAP, 7 (7.4%) died or were discharged within two days and 20 (21.3%) had a VAP diagnosis (FDA or physician diagnosis) during the weekend. The most common diagnostic sample type was endotracheal aspirate (293/359 patients, 81.6%, of which 72% had a quantitative culture), the most common pathogens were *Staphylococcus aureus* (94/359, 26.2%), *Haemophilus influenzae* (58, 16.2%) and *Pseudomonas aeruginosa* (54, 15.0%) (See Supplementary Table 4 for pathogen distribution per sample type). Of the MBE VAP patients 238/359 (66.3%) had only one relevant VAP pathogen identified, while 121 (33.7%) had at least two. *S. aureus* and *H. influenzae* were commonly found together (19 patients). Conversely, *P. aeruginosa* was mostly recorded as a single pathogen (33/54 patients).

The dominant VAP strain for ICUs from Spain, France, Belgium and Czech Republic was *S. aureus*, while for Romania, Serbia and Croatia, it was *Acinetobacter* species (Supplementary Table 5). In addition, *H. influenzae* seemed to be more common in patients with early VAP, whereas *P. aeruginosa* tended to be more common in patients with late VAP (Supplementary Table 6).

Based on strains tested, 14/77 (18.2%) patients with *S. aureus* had methicillin-resistant (MRSA) strains and 1/43 (2.3%) of *H. influenzae* were third generation cephalosporin resistant. For *P. aeruginosa*, 10/46 (21.7%) patients had strains which were resistant to ceftazidime, and 8/47 (17.0%) to carbapenems. It is important to note that not all strains were tested for all relevant resistance types. For example, 17 and 28 patients of 94 with *S. aureus* did not have their strains tested for methicillin and vancomycin resistance, respectively (Supplementary Table 7).

### Clinical outcomes

The cumulative incidence of ICU mortality was 34.2% (199/581, 95% CI 30.4%−38.0%) in the VAP population, compared to 29.3% (836/2854, 95% CI 27.6%−30.9%) in the non-VAP population (Table 3, Supplementary Fig. 3).

In the VAP population, the median overall length of ICU stay was 24 days (IQR 15–36), of which 6.5 days (IQR 4–10) were before VAP diagnosis and 15 days (IQR 8–27) were after VAP diagnosis. In contrast, the median overall length of ICU stay was 11 days (IQR 7–19) for the non-VAP population (*n* = 2854 with non-missing data).

For VAP patients, the median overall time under IMV until first extubation was 17 days (IQR 11–29), of which 6 days (IQR 4–10) were before VAP diagnosis and 10 days (IQR 6–20) were after VAP diagnosis. In comparison, the non-VAP patients had a median of 7 days (IQR 4–12) until first extubation (*n* = 2855 with non-missing data).

Of 574 VAP patients with complete data, 248 (43.2%) had zero IMV free days up to 28 days after VAP onset, this was due to death in 175/248 patients (70.6%). While 326/574 VAP patients (56.8%) had a median of 21 IMV free days (IQR 13–24) (Supplementary Fig. 4).

We investigated three definitions of clinical cure between days 7–10 (Supplementary Table 1) [26]. For the first definition, 363 VAP patients could be assessed (115 patients were not present in the ICU at day 7 and 112 patients had missing data) and 46 (12.7%, 95% CI 9.4%−16.5%) met the clinical cure definition. By labelling patients who died before day 10 as not cured and those who were discharged alive by day 10 as cured, (definition 3), clinical cure increased to 144/523 (27.5%, 95% CI 23.7%−31.6%) (Table 3). Different restrictions of the VAP population had very little effect on clinical cure proportions (Supplementary Table 8).

For microbiological cure, in the MBE VAP population, also three definitions were explored (Supplementary Table 1). For the first definition, 267 patients could not be assessed (30 patients did not have the same type of sample assessed at VAP diagnosis and test of cure visit, 63 patients were not present in the ICU at day 7 and 174 patients had missing data), reducing to 145 for definition 3, when considering patients who died by day 10 as not cured and patients who were discharged alive by day 10 as cured. Microbiological cure increased from 47.8% (95% CI 37.3%−54.5%) (definition 1) to 50.9% (95% CI 44.0%−57.8%) (definition 3, Table 3).

**Table 3:** Clinical outcomes for the VAP population (*N* = 590) and MBE VAP population (*N* = 359)

Clinical outcome	VAP (*N* = 590)
Vital status at ICU discharge*	
Dead Alive Missing	199 (34.2%: 30.4–38.0) 382 (65.8%: 61.9–69.7) 9
Vital status at 28 days after VAP*	
Dead Alive Missing	175 (30.3%: 26.6–34.1) 397 (69.7%: 65.9–73.4) 18
*Overall length of ICU stay* (days)	
Median (IQR) Missing	24 (15–36) 9
*Length of ICU stay before VAP* (days)	
Median (IQR)	6.5 (4–10)
*Length of ICU stay after VAP* (days)	
Median (IQR) Missing	15 (8–27) 9
*Overall length of IMV until first extubation* (days)	
Median (IQR) Missing	17 (11–29) 9
*Length of IMV before VAP* (days)	
Median (IQR)	6 (4–10)
*Length of IMV after VAP* (days)	
Median (IQR) Missing	10 (6–20) 9
*IMV-free days within 28 days after VAP diagnosis*	
Median (IQR): for all Median (IQR): for patients with IMV-free > 0 Missing	8.5 (0–22) 21 (13–24) 16
*Clinical Cure 1* n (%)	
Cured Not Cured Total Left ICU Missing	46 (12.7) 317 (87.3) 363 (100) 115 112
*Clinical Cure 2* n (%)	
Cured Not Cured Total Left ICU Missing	46 (10.2) 403 (89.8) 449 (100) 47 94
*Clinical Cure 3* n (%)	
Cured Not Cured Total Left ICU Missing	144 (27.5) 379 (72.5) 523 (100) 0 67
*Microbiological Cure 1* n (%)	
Cured Not Cured Total Not same sample Left ICU Missing	44 (47.8) 48 (52.2) 92 (100) 30 63 174
*Microbiological Cure 2* n (%)	
Cured Not Cured Total Not same sample Left ICU Missing	43 (29.1) 105 (70.9) 148 (100) 29 23 159
*Microbiological Cure 3* n (%)	
Cured Not Cured Total Not same sample Left ICU Missing	109 (50.9) 105 (49.1) 214 (100) 27 0 118

*Data are given as n (cumulative incidence: 95% confidence intervals). Data are crude, unadjusted values

### Antibiotic use

Among patients with FDA and physician VAP within 1 day, 375/436 (86.0%) received VAP-related antibiotics. Of 61 patients without VAP-related antibiotics, 52 patients started antibiotic therapy more than 1 day before/after their physician VAP diagnosis. Nine patients did not receive VAP-related antibiotic therapy; 4 (44.4%) had no or non-VAP microorganisms present in their microbiological sample(s), 1 (11.1%) patient was under treatment limitation, and 1 (11.1%) patient died the day of their physician VAP diagnosis, for three patients, reasons were not documented. Antibiotics prescribed in the ABT VAP population varied substantially by country. The most frequently prescribed initial antibiotic was piperacillin-tazobactam (68/375, 18.1%), followed by meropenem 67/375 (17.9%) (Supplementary Fig. 5, Supplementary Table 9). The number of patients started on combination therapy was 70/375 (18.7%), and the most common combinations were linezolid and meropenem (10/70, 14.3%) and colistin and meropenem (7/70, 10.0%).

## Discussion

We present the results of the warm-base network, POS-VAP, including 25 ICUs across 11 European countries, recruiting 3,446 patients at risk of VAP with 590 confirmed VAP cases (cumulative incidence 17.1%), defined using standardised criteria. Implementation of prevention strategies was heterogeneous across countries, with head-of-bed elevation applied most often. The three most frequently identified pathogens were *S. aureus*,* H. influenzae* and *P. aeruginosa.* High carbapenem resistance was reported for *Klebsiella* and *Acinetobacter* species, which were especially frequent in the Serbian and Romanian sites, explaining the frequent use of colistin reported there. Mortality, length of ICU stay and duration of IMV among VAP patients remain high, while VAP clinical cure proportions were heavily influenced by definitions used, but were low.

The cumulative incidence of VAP found within POS-VAP, falls within the range reported in the literature (4–40% [3, 4]). The heterogeneous VAP incidences reported previously highlight the need for a standardised VAP definition [14]. Within POS-VAP, harmonised criteria were used to diagnose VAP based on the US FDA definition. Importantly, these criteria may not catch all VAP episodes and may identify false-positive episodes [27]. The use of an adjudication committee should be considered in trials with VAP as an outcome, but this was not feasible in the current observational study. Nevertheless, in our study FDA VAP aligned well with physician diagnosis. While US FDA criteria are not often used in observational studies, they are applied in interventional clinical trials and provide crucial information on baseline VAP rates for their design. Using a harmonised definition, we compared incidences across countries, showing heterogeneity in VAP cumulative incidence (range: 7.6% (Croatia)-29.6% (Romania) for countries recruiting at least 100 patients). This could be caused by differences in VAP epidemiology, differences in diagnostic work-up between sites (e.g. chest X-ray frequency) or differences in the implementation of prevention measures. It underlines the importance of benchmarking between sites to highlight outliers and incentivise improvements to patient care. It also shows the utility of site-specific data to inform the design and planning of interventional or observational studies. Further in-depth analyses are planned to better understand the potential risk factors associated with VAP development such as age, sex and reason for ICU admission.

Microbiological confirmation of VAP is complex, frequently no pathogens or only colonising pathogens are detected. In studies on HAP/VAP, 69.5%−85.4% of patients had a causative pathogen identified [28, 29]. In POS-VAP, microbiological documentation was available in 60.7% of the VAP patients, which is lower and may be associated with reliance on routine procedures. As many reports combine VAP and HAP, direct comparison of species and antibiotic resistance rates from POS-VAP to previous studies is difficult [30]. Nevertheless, the EU-VAP/CAP study, which investigated the outcome of nosocomial pneumonia (HAP and VAP), found *S. aureus* to be the dominant isolate for Spain, France and Belgium, similarly to POS-VAP. For Italy and Portugal, the most commonly reported isolate was *P. aeruginosa*, while it was *Acinetobacter* in Greece and Turkey [28]. Furthermore, SENTRY, which monitors bacterial isolates from patients hospitalised with pneumonia (HAP, VAP and hospitalised CAP) [31], shows similar results to POS-VAP, indicating that *P. aeruginosa*, *S. aureus* and *E. coli* are the most common pathogens in western Europe, while *P. aeruginosa*, *K. pneumoniae* and *A. baumannii* are the most common pathogens in eastern Europe. Reported resistance proportions overlapped as well, with lower proportions across western than eastern European countries [31]. Multidrug-resistant isolates are often more commonly recorded in Eastern and Southern Europe than Western and Northern Europe [32]. This has been associated with more frequent use of broad-spectrum antibiotics and differences in infection control practices in Eastern and Southern Europe [33, 34]. Compared to SENTRY and the EU-VAP/CAP study, POS-VAP has strict patient in- and exclusion criteria, used harmonised definitions for VAP diagnosis and standardised prospective data collection for microbiological results across sites, with a clear focus on the ICU population, improving validity of the results.

Mortality was higher in the VAP population compared to non-VAP patients, although, the confidence intervals overlapped. This increased mortality could be a consequence of VAP itself, or a reflection of the higher baseline vulnerability of this population, increasing both the risk for VAP and death. It is difficult to assess the mortality attributable to VAP due to time-varying confounding, literature estimates range between 0% and 27% [5–9]. Several VAP prevention trials show a decrease in VAP incidence, but no improvements in other clinical outcomes (mortality, length of ICU stay or duration of IMV) [35, 36]. We found that VAP patients had a longer ICU length of stay and duration of IMV than non-VAP patients, although no conclusions can be drawn based on these descriptive analyses. Future in-depth analyses are planned to better understand the causal effect of VAP on mortality, ICU length of stay and IMV duration in the current cohort.

There is no clear standard for endpoints used in interventional trials assessing VAP treatment. Common endpoints include mortality, duration of ICU stay and IMV, or clinical/microbiological cure. We have provided baseline data for these endpoints. Although mortality is objective, the data show that baseline mortality for IMV patients is high irrespective of VAP, making it difficult to demonstrate an impact [37]. Clinical cure is subjective, and the consensus definition applied in POS-VAP [26] resulted in very low clinical cure proportions, even though a large proportion of patients were discharged alive. The consensus definition included the resolution of *all* signs/symptoms present at VAP onset (Supplementary Table 2) and the improvement or lack of progression of radiological signs of pneumonia; some of these symptoms may be too general for ICU patients to be used for VAP cure. Several different definitions of clinical cure are found in the literature [14]. For example, Kollef et al. [38] defined clinical cure as absence of bacterial growth and Clinical Pulmonary Infection Score ≤ 6 by day 14, showing a similarly low clinical cure of 23%. Another study [39], showed a clinical cure of 46% in patients with carbapenem-resistant *A. baumannii* VAP, where cure was defined as resolution of fever or hypothermia, disappearance of tracheal secretions with leukocyte counts < 25 cells/mm^3^, PaO_2_/FiO_2_ ratio >240 or IMV no longer needed, and partial or total resolution of respiratory crackles. This variation clearly shows the importance of harmonising definitions across studies.

The high burden of VAP highlights the need for trials evaluating new preventive or treatment strategies. POS-VAP could serve as a warm-base for future trials, as it is the largest prospective cohort focused on VAP in Europe, and patient recruitment is ongoing with an average rate of 2.5 patients/site/week. Embedding a study within POS-VAP would increase efficiency by decreasing time to first patient enrolment, aligning site selection with study inclusion criteria, and providing data for well-informed estimates of the required sample size [15].

Our study has several limitations. As it has gathered routinely collected clinical and microbiological results, some data was missing. Not all sites had a detailed screening log, and while the reported reasons for exclusion of eligible patients do not seem to have introduced selection bias, this cannot be confirmed for sites without a screening log. We also relied on routine, clinically driven screening for VAP, which might have resulted in underreporting of VAP in certain sites. Moreover, unsuccessful extubations (patients being re-intubated within 48 h) were not tracked. In addition, some sites did not collect microbiological samples within the strict time window (2 days before/after FDA VAP criteria were met). Furthermore, different approaches to consent have been applied at site/country level (informed consent, waiver of consent, hospital wide consent and non-objection notice), which may have affected the benchmarking of results between centres. In general, the requirement of consent may reduce the external validity of results but was crucial to allow re-use of this rich data source. In addition, the true compliance to the VAP prevention measures is unknown due to these only being recorded at intubation without follow-up over time. Furthermore, recruitment was unequal across sites and countries, due to sites opening at different times, and some countries may have been overrepresented, therefore results have also been presented per country. Finally, although we have included sites from 11 different countries, there are specific European regions which are not represented, like the Scandinavian and Baltic regions. We hope to enrich the POS-VAP network by including these regions in future cohort analyses.

An important strength of our study is the multi-centre approach across several European countries. In addition, the presented data are available for re-use, and study metadata is available [40]. The perpetual nature of this study will allow the cohort to grow and provide up-to-date information about VAP epidemiology and aetiology.

## Conclusions

In conclusion, among critically ill patients in European the incidence of VAP remains substantial with large heterogeneity across countries, associated with poor clinical prognosis. Furthermore, POS-VAP has shown a large variation in strategies for VAP prevention and treatment and VAP aetiology across countries, with methicillin-susceptible *S. aureus* being the most common causative pathogen, especially in Northern and Western Europe. The data presented here should inform and support future interventional studies that are required to improve the prevention, management and outcomes of mechanically ventilated patients.

## Supplementary Information

Supplementary Material 1.

## Data Availability

The data analysed within is from the prospective, observational POS-VAP study and are not publicly available. However, data access for secondary analyses can be requested from the POS-VAP scientific coordinators (BF, MEAdK, and CHvW).

## Abbreviations

ICU: Intensive Care Unit
IMV: Invasive Mechanical Ventilation
VAP: Ventilator-Associated Pneumonia
CT: Computed Tomography
POS: Perpetual Observational Study
Ecraid: European Clinical Research Alliance on Infectious Diseases
GCP: Good Clinical Practice
MBE: MicroBiologically Evaluable
ABT: AntiBiotic Treated
FDA: Food and Drug Administration
PaO2/FiO2: Ratio of Partial pressure of Oxygen in arterial blood to the Fraction of Inspiratory Oxygen concentration
MAP: Mean Arterial Pressure
BAL: Broncho-Alveolar Lavage
ETA: EndoTracheal Aspirates
APACHE: Acute Physiology And Chronic Health Evaluation
SOFA: Sequential Organ Failure Assessment
IQR: Inter-Quartile Range
CIF: Cumulative Incidence Functions
CI: Confidence Interval
AB: AntiBiotic
CVA: CerebroVascular Accident
TIA: Transient Ischemic Attack
SARS-CoV: Severe Acute Respiratory Syndrome Corona Virus
GI: GastroIntestinal
CNS: Central Nervous System
STEMI: ST Elevated Myocardial Infarction
NSTEMI: Non-ST Elevated Myocardial Infarction
COVID: Corona Virus
MRSA: Methicillin Resistant *Staphylococcus Aureus*
ESBL: Extended-Spectrum Beta-Lactamase
