# Supplementary material for: Perpetual observational study of the clinical and microbiological epidemiology of ventilator-associated pneumonia in Europe
Source: Crit Care. 2026 Mar 12;30:112. doi: 10.1186/s13054-025-05753-5 (PMC12980874; doi:10.1186/s13054-025-05753-5)
Supplement: Supplementary file 1 — Supplementary Material 1. [file 13054_2025_5753_MOESM1_ESM.docx]

**Supplementary Materials**

**Perpetual observational study on the clinical and microbiological epidemiology of ventilator-associated pneumonia in Europe**

Holly Jackson^1^, Ana Catalina Hernandez Padilla^2^, Lisanne E.M. Vintcent^3^, Aleksandra Barac^4^, Olaf Cremer^3,5^, Thomas Daix^2^, Jan J. De Waele^6,7^, Lorena Forcelledo^8,9^, Olivier Barraud^10^, Marc J.M. Bonten^3,11^, Stephan Harbarth^1^, Bruno Francois^2^, C.H. (Henri) van Werkhoven^3^, Marlieke E. A. de Kraker^1^

On behalf of the POS-VAP Study Group and the ECRAID-Base Consortium

**Corresponding Author:** Holly Jackson, [holly.jackson@hug.ch](mailto:holly.jackson@hug.ch),
1. Infection Control Program, Geneva University Hospitals and Faculty of Medicine, World Health Organization Collaborating Center, Geneva, Switzerland

**Methods**

**Supplementary Table 1 Patients recruitment per country for the at-risk population (N=3,446)**


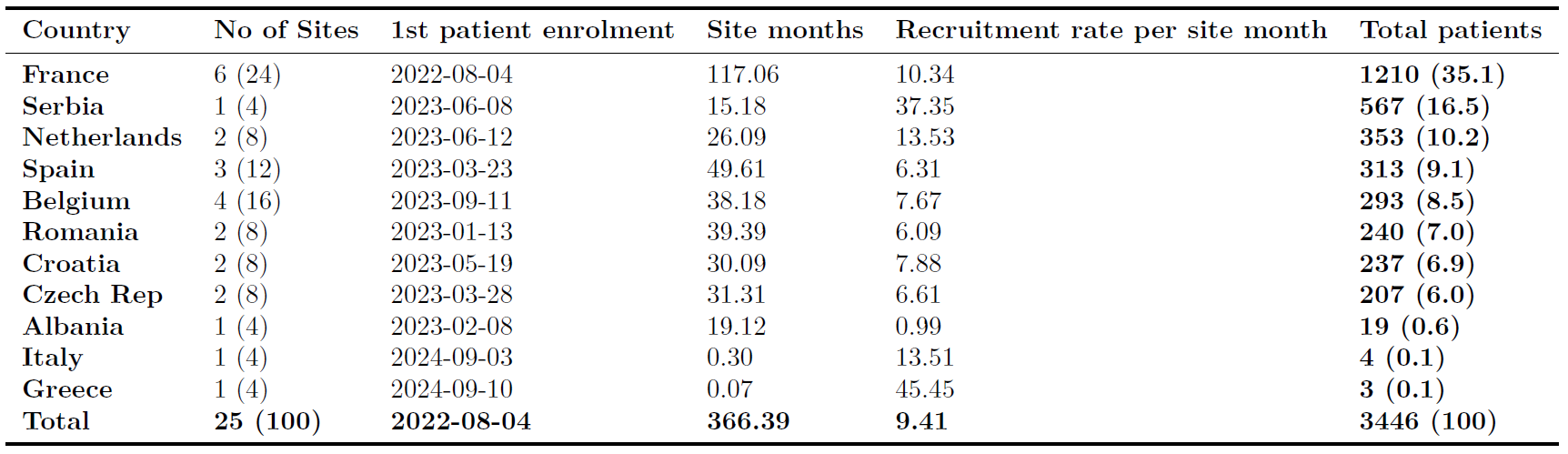


Count data are given as n (column %), Site months is the sum of the number of months all sites within a country were open for, Recruitment rate is the total number of patients divided by the site months per country

**Supplementary Table 2 Definitions for study endpoints**

| ***Definitions*** |
| --- |
| **Ventilator associated pneumonia** **(VAP)**  FDA criteria were used to diagnose VAP; these apply to any patient under IMV for at least 48 hours and up to 48 hours after extubation. Only VAP during the first ventilation episode were recorded, where the first ventilation episode ends at date of death, discharge or 48 hours after successful extubation (without re-intubation), whichever comes first. VAP is diagnosed by the presence of at least one clinical respiratory feature (pulmonary symptoms, hypoxemia or worsening of PaO2/FiO2 ratio, need for acute changes in the ventilator support system or new onset of suctioned respiratory secretions), at least one systemic sign (fever or hypothermia, leucocytosis or leukopenia, and increased immature neutrophils), and a chest imaging (Chest X-ray / CT scan) showing the presence of new or progressive infiltrate(s) suggestive of bacterial pneumonia [18]. |
| **Physician diagnosis of VAP**  The attending physician diagnosed the patient as having a VAP. |
| **Invasive mechanical ventilation-free-days within 28 days after VAP diagnosis**  Day 0 was defined as the date of VAP diagnosis and IMV-free days were counted from day 1 to day 28. IMV free days were defined as 0 if the patient required IMV for the whole 28 days after VAP diagnosis, or if the patient died within 28 days of their VAP diagnosis. If the patient was successfully extubated from IMV (removed from IMV for at least 48 hours consecutively without being re-intubated) at any point within 28 days of their VAP diagnosis, then their IMV free days were calculated as 28-x where x is the total number of days that IMV was required (including if the patient experienced multiple periods of IMV). If a patient was discharged from ICU in this time, they were considered not ventilated on their date of discharge and thereafter. |
| **Clinical cure  Definition 1**: Clinical cure was defined as a combination of:   1. resolution of signs and symptoms present at VAP onset (i.e., back to the level prior to VAP onset), 2. the resolution of hypotension (MAP< 70 mmHg or need for vasopressor support) if present at VAP onset and 3. improvement or lack of progression of radiological signs of pneumonia [26].   Patients had to meet all three criteria to be labelled as ‘clinically cured’. Any patient who did not meet at least one of the above criteria were labelled as ‘not clinically cured’.  Clinical cure was assessed between days 7-10 after the date of VAP onset. Those patients who did not have their symptoms assessed (either at all or in the appropriate time window), even though they were present in the ICU at least until day 7, were categorised as ‘Missing’. Patients who had been discharged alive from the ICU or who died before day 7 were categorised as ‘left ICU’.  Sensitivity analyses were performed considering the following:  **Definition 2**: Patients had to meet the above three criteria to be labelled as ‘clinically cured’. Any patient who did not meet at least one of the three criteria was categorised as ‘not clinically cured’. In addition, all patients who died before or on day 10 after VAP onset were classified as ‘not clinically cured’.  **Definition 3**: Patients had to meet the above three criteria to be labelled as ‘clinically cured’. Furthermore, all patients who were discharged alive before or on day 10 after VAP onset were also categorised as ‘clinically cured’. Any patient who did not meet at least one of the three criteria was labelled as ‘not clinically cured’. In addition, all patients who died before or on day 10 after VAP onset were classified as ‘not clinically cured’. However, any patient who was discharged alive and who died before or on day 10 after VAP onset was labelled as ‘not clinically cured’.  Definitions 2 and 3 will therefore include fewer patients labelled as ‘missing’ and ‘left ICU’.  Furthermore, we investigated how the proportion of patients with clinical cure changed for each of the three definitions, for three restrictions on the VAP population including: 1) all VAP patients with a physician VAP diagnosis, 2) all VAP patients with a physician VAP diagnosis +/-1 day form their FDA VAP diagnosis and who started antibiotic treatment within +/-1 day from their physician VAP diagnosis, and 3) all VAP patients with a microbiological sample taken +/-2 days from their FDA VAP diagnosis. |
| **Microbiological cure Definition 1**: Microbiological cure was assessed only for the MBE VAP population. Microbiological cure is defined as the absence of the microorganism responsible for pneumonia in the same type of sample as the one which was used to identify their VAP causative microorganism (broncho- alveolar lavage (BAL), blood culture, endotracheal aspirates (ETA) or sputum sample).  Microbiological cure was assessed between days 7-10 after the date of VAP onset. Those patients with different types of samples for VAP diagnosis and VAP cure, were labelled as ‘Not same sample’. Any patient who did not have their microbiological cure assessed (either at all or between days 7-10 after their VAP diagnosis), even though they were present in the ICU until at least day 7 were categorised as ‘Missing’. Finally, we categorised patients who had been discharged alive from the ICU or who died before day 7 as ‘left ICU’.  Sensitivity analyses were performed considering the following:  **Definition 2**: Patients had to have the microorganism responsible for their pneumonia absent in the same type of sample as the one which was used to identify their VAP causative microorganism, to be labelled as ‘microbiologically cured.’ Any patient who did have the same microorganism present in the same type of sample was categorised as ‘not microbiologically cured.’ In addition, we classified all patients who died before or on day 10 after VAP onset as ‘not microbiologically cured’.  **Definition 3**: Patients had to have the microorganism responsible for their pneumonia absent in the same type of sample as the one which was used to identify their VAP causative microorganism, to be labelled as ‘microbiologically cured.’ Furthermore, all patients who were discharged alive before or on day 10 after VAP onset were categorised as ‘microbiologically cured’. Any patient who did have the same microorganism present in the same type of sample which was used to identify their VAP causative pathogen was labelled as ‘not microbiologically cured.’ Additionally, all patients who died before or on day 10 after VAP onset were classified as ‘not microbiologically cured’. However, any patient who was discharged alive and who died before or on day 10 after VAP onset was labelled as ‘not microbiologically cured’.  Definitions 2 and 3 will therefore include fewer patients labelled as ‘not same sample’, ‘missing’ and ‘left ICU’. |

**Supplementary Table 3 Antibiotic treatment classes**

| **Antibiotic class** | **Antibiotic treatment** |
| --- | --- |
| Aminoglycosides | amikacin, gentamicin, tobramycin |
| Anti-MRSA agent | linezolid, vancomycin |
| Beta-lactams (anti-*Pseudomonas aeruginosa* activity) | aztreonam, cefepime, ceftazidime, ceftazidime and beta-lactamase inhibitor, ceftolozane and beta-lactamase inhibitor, imipenem and cilastatin, meropenem, piperacillin, piperacillin and beta-lactamase inhibitor |
| Beta-lactams (no anti- *Pseudomonas aeruginosa* activity) | amoxicillin, amoxicillin and beta-lactamase inhibitor, ampicillin, ampicillin and beta-lactamase inhibitor, benzylpenicillin, cefotaxime, ceftriaxone, cefuroxime, cloxacillin, ertapenem, flucloxacillin, oxacillin |
| Colistin | colistin |
| Fluoroquinolones (anti- *Pseudomonas aeruginosa* activity) | ciprofloxacin, levofloxacin |
| Others | chloramphenicol, clindamycin, clarithromycin, cotrimoxazole, daptomycin, erythromycin, fosfomycin, metronidazole, moxifloxacin, sulfadiazine and trimethoprim, sulfamethoxazole and trimethoprim, Sulfonamides combinations (excl. trimethoprim), tigecycline |

To create the stacked probability plot in the main text, we utilised the extended illness-death model displayed in Supplementary Figure 1. Within POS-VAP, patients enter the study when they are at risk of developing ventilator-associated pneumonia (VAP) (under invasive mechanical ventilation (IMV) for at least 48 hours). While at risk of VAP, they are also at risk of several competing events: extubation (being removed from the ventilator without re-intubation for at least 48 hours), ICU discharge without extubation, and ICU death without extubation. When a patient develops VAP, they are still at risk of extubation, ICU discharge without extubation, and ICU death without extubation. Finally, if the patient has been successfully extubated (either with or without VAP) they are then at risk of ICU death and ICU discharge.

**Supplementary Figure 1 Extended illness-death model incorporating intermediate states: VAP and extubation**

**
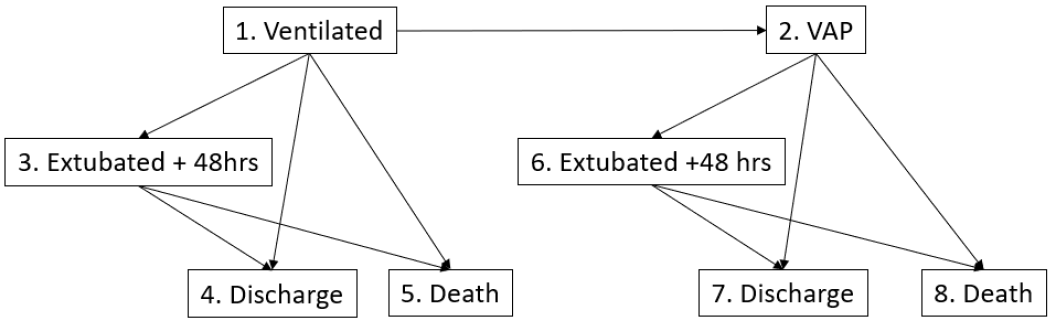
**

States are displayed in the boxes and transitions are represented by the arrows

We allow the probability of patients transitioning between states to vary over time. The POS-VAP data is used to estimate the probability that a patient makes each transition at each time point over the course of the total follow-up time. Time zero is 48 hours after first intubation. The longest follow-up time a patient had was 255 days and the median (IQR) follow-up time was 10 (5, 20) days. Finally, the follow-up time in the stacked probability plot is counted in full day intervals.

**Results**

**Supplementary Figure 2 Cumulative incidence of VAP and VAP incidence rate per 1000 ventilator days per country for the at-risk population (N=3,446)**


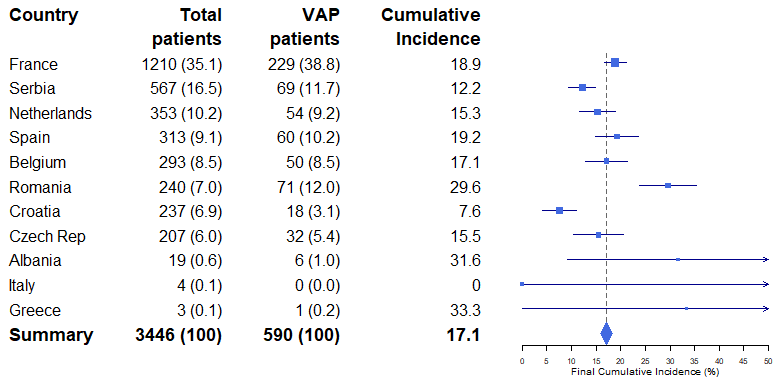

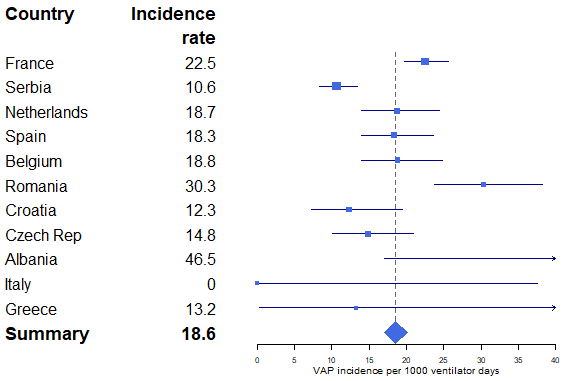


Count data are given as n (% of patients), cumulative incidence (%) accounts for competing events (extubation +48H, discharge, death) and incidence rate is per 1000 ventilator days

**Supplementary Table 4 Pathogens identified per sample type in the microbiologically evaluable (MBE) VAP population (N=359)**

**
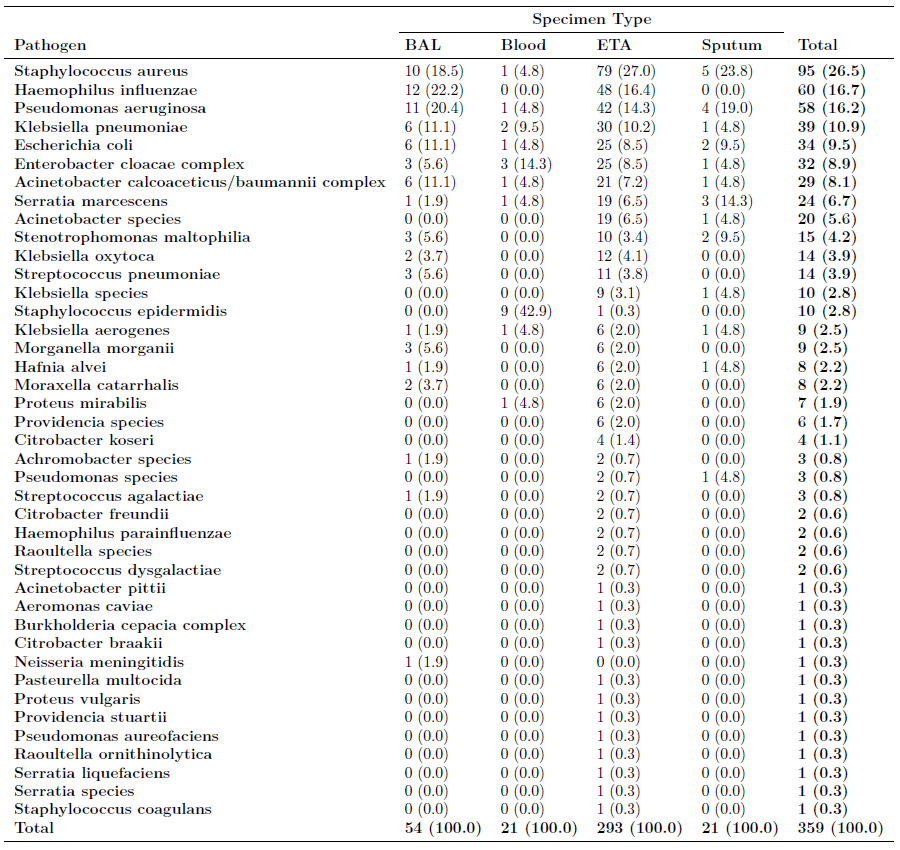
**

BAL, Broncho-Alveolar Lavage; ETA, EndoTracheal Aspirates
Data are given as n (% of patients). Patients can be counted multiple times, if they had more than one positive sample of different type, or multiple pathogens identified in the same sample type

To note, of the 293 patients with positive ETA samples, 211 (72.0%) had a quantitative culture performed. Of the 54 patients with positive BAL samples, 29 (53.7%) had a quantitative culture performed. Of the 21 patients with positive sputum samples, 2 (9.5%) had a quantitative culture performed.

**Supplementary Table 5 Pathogens identified per patient in the total VAP population (N=590), stratified by country**


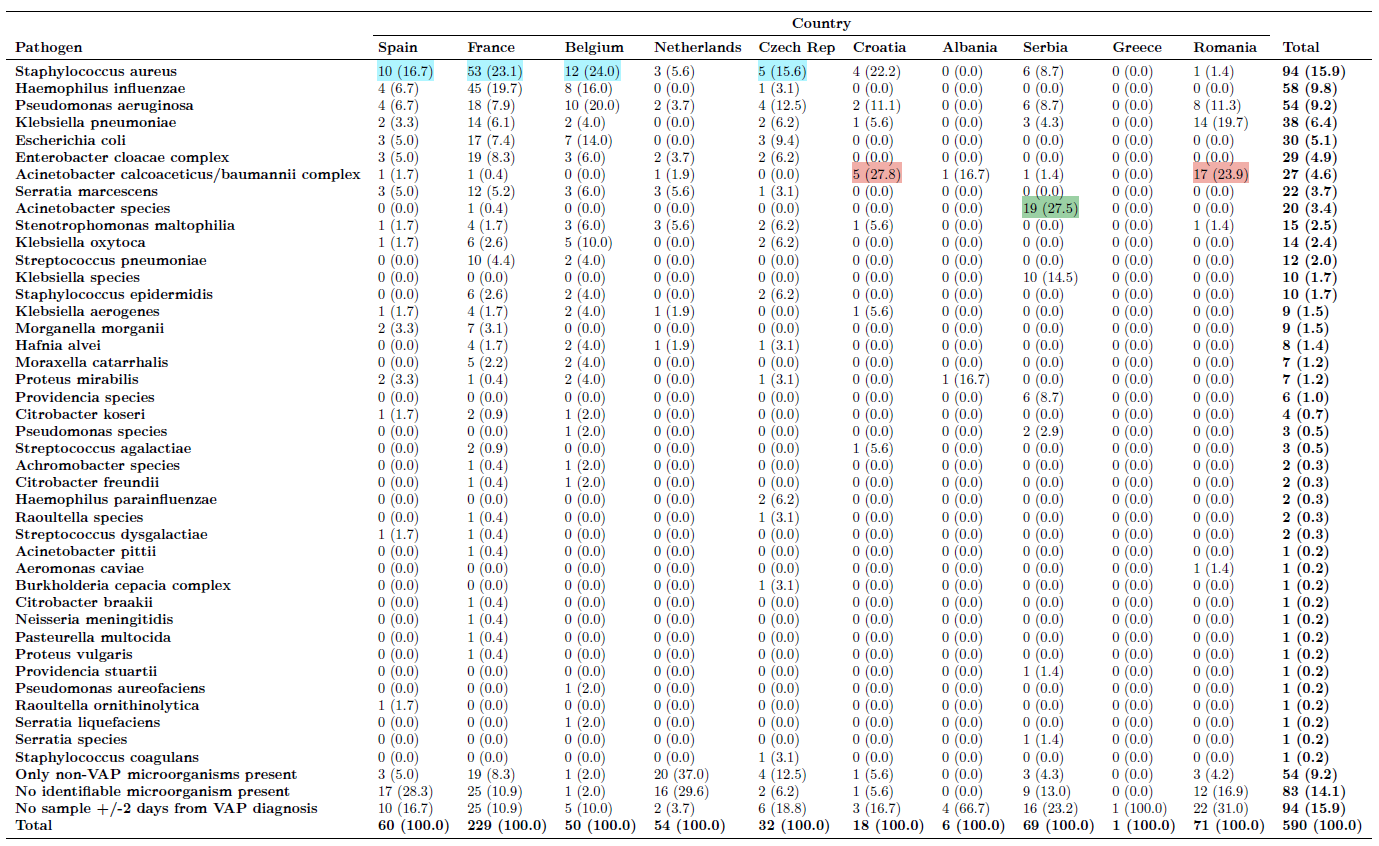

*The dominating pathogen per country is highlighted, where each pathogen is highlighted with a different colour

**Supplementary Table 6 Pathogens identified per patient in the MBE VAP population (N=359), stratified by early vs late onset VAP**

**
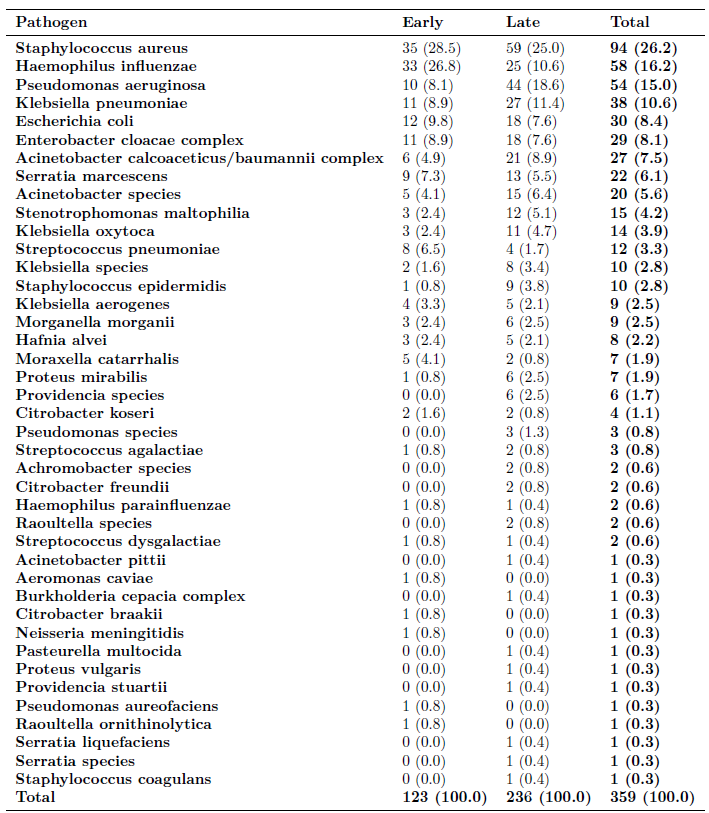
***Early VAP is defined as VAP diagnosed $\leq$ 4 days after intubation; late VAP is diagnosed > 4 days from intubation

**Supplementary Table 7 Selected resistance profiles of VAP pathogens identified in the microbiologically evaluable (MBE) VAP population (N=359) at patient level**

| **Resistance** | **Tested/Total MBE VAP population** | **No. Resistant (%)** |
| --- | --- | --- |
| ***Achromobacter* species**  Carbapenem  ***Acinetobacter calcoaceticus/baumannii complex***  Carbapenem | 1/2  26/27 | 0 (0.0)  22 (84.6) |
| ***Acinetobacter pittii***  Carbapenem  ***Acinetobacter* species**  Carbapenem  ***Burkholderia cepacia complex***  Carbapenem 3^rd^ generation cephalosporins  ***Citrobacter braakii***  Carbapenem  3^rd^ generation cephalosporins  ESBL  ***Citrobacter freundii***  Carbapenem  3^rd^ generation cephalosporins  ESBL  ***Citrobacter koseri***  Carbapenem  3^rd^ generation cephalosporins  ESBL  ***Enterobacter cloacae complex***  Carbapenem  3^rd^ generation cephalosporins  ESBL  ***Escherichia coli***  Carbapenem  3^rd^ generation cephalosporins  ESBL  ***Haemophilus influenzae***  3^rd^ generation cephalosporins  ***Haemophilus parainfluenzae***  3^rd^ generation cephalosporins  ***Hafnia alvei***  Carbapenem  3^rd^ generation cephalosporins  ESBL  ***Klebsiella aerogenes***  Carbapenem  3^rd^ generation cephalosporins  ESBL  ***Klebsiella oxytoca***  Carbapenem  3^rd^ generation cephalosporins  ESBL  ***Klebsiella pneumoniae***  Carbapenem  3^rd^ generation cephalosporins  ESBL  ***Klebsiella* species**  Carbapenem  3^rd^ generation cephalosporins  ESBL  ***Moraxella catarrhalis***  3^rd^ generation cephalosporins  ***Morganella morganii***  Carbapenem  3^rd^ generation cephalosporins  ESBL  ***Neisseria meningitidis***  3^rd^ generation cephalosporins  ***Pasteurella multocida***  Carbapenem  3^rd^ generation cephalosporins  ESBL  ***Proteus mirabilis***  Carbapenem  3^rd^ generation cephalosporins  ESBL  ***Proteus vulgaris***  Carbapenem  3^rd^ generation cephalosporins  ESBL  ***Providencia* species**  Carbapenem  3^rd^ generation cephalosporins  ESBL  ***Providencia stuartii***  Carbapenem  3^rd^ generation cephalosporins  ESBL  ***Pseudomonas aeruginosa***  Carbapenem  Ceftazidime  ***Pseudomonas aureofaciens***  Carbapenem  3^rd^ generation cephalosporins  ***Pseudomonas* species**  Carbapenem  3^rd^ generation cephalosporins  ***Raoultella ornithinolytica***  Carbapenem  3^rd^ generation cephalosporins  ESBL  ***Raoultella* species**  Carbapenem  3^rd^ generation cephalosporins  ESBL  ***Serratia liquefaciens***  Carbapenem  3^rd^ generation cephalosporins  ESBL  ***Serratia marcescens***  Carbapenem  3^rd^ generation cephalosporins  ESBL  ***Serratia* species**  Carbapenem  3^rd^ generation cephalosporins  ESBL  ***Staphylococcus aureus***  Methicillin  Vancomycin  ***Stenotrophomonas maltophilia***  3^rd^ generation cephalosporins  ***Streptococcus pneumoniae***  3^rd^ generation cephalosporins | 1/1  16/20  0/1 0/1  1/1  1/1  0/1  2/2  1/2  1/2  3/4  4/4  1/4  26/29  23/29  18/29  26/30  28/30  18/30  43/58  1/2  7/8  4/8  2/8  7/9  6/9  7/9  9/14  13/14  4/14  37/38  33/38  26/38  4/10  4/10  0/10  3/7  9/9  9/9  9/9  0/1  0/1  1/1  0/1  4/7  5/7  4/7  1/1  1/1  0/1  4/6 4/6 0/6  1/1  1/1  0/1  47/54  46/54  1/1  1/1  2/3  2/3  1/1  1/1  1/1  1/2 1/2 2/2  1/1  1/1  0/1  19/22  18/22  12/22  1/1  1/1  0/1  77/94  66/94  5/15  9/12 | 0 (0.0)  15 (93.8)  - -  0 (0.0)  0 (0.0)  -  0 (0.0)  1 (100.0)  0 (0.0)  0 (0.0)  0 (0.0)  0 (0.0)  1 (3.8)  16 (69.6)  8 (44.4)  1 (3.8)  2 (7.1)  2 (11.1)  1 (2.3)  0 (0.0)  0 (0.0)  1 (33.3)  0 (0.0)  0 (0.0)  3 (50.0)  1 (14.3)  0 (0.0)  0 (0.0)  0 (0.0)  13 (35.1)  18 (54.5)  8 (30.8)  4 (100.0)  4 (100.0)  -  0 (0.0)  0 (0.0)  3 (33.3)  0 (0.0)  -  -  0 (0.0)  -  1 (25.0)  1 (20.0)  0 (0.0)  0 (0.0)  0 (0.0)  -  3 (75.0)  4 (100.0) -  1 (100.0)  1 (100.0) -  8 (17.0) 10 (21.7)  0 (0.0)  1 (100.0)  0 (0.0) 1 (100.0)  0 (0.0)  0 (0.0)  0 (0.0)  0 (0.0)  0 (0.0)  0 (0.0)  0 (0.0)  0 (0.0)  -  0 (0.0)  6 (33.3)  2 (16.7)  1 (100.0)  0 (0.0)  -  14 (18.2)  0 (0.0)  2 (40.0)  0 (0.0) |

ESBL, extended-spectrum beta-lactamase

**Supplementary Figure 3 Cumulative incidence of ICU mortality and discharge alive from ICU in A) non-VAP patients N=2,856 and B) VAP patients N=590 with 95% confidence intervals**

**
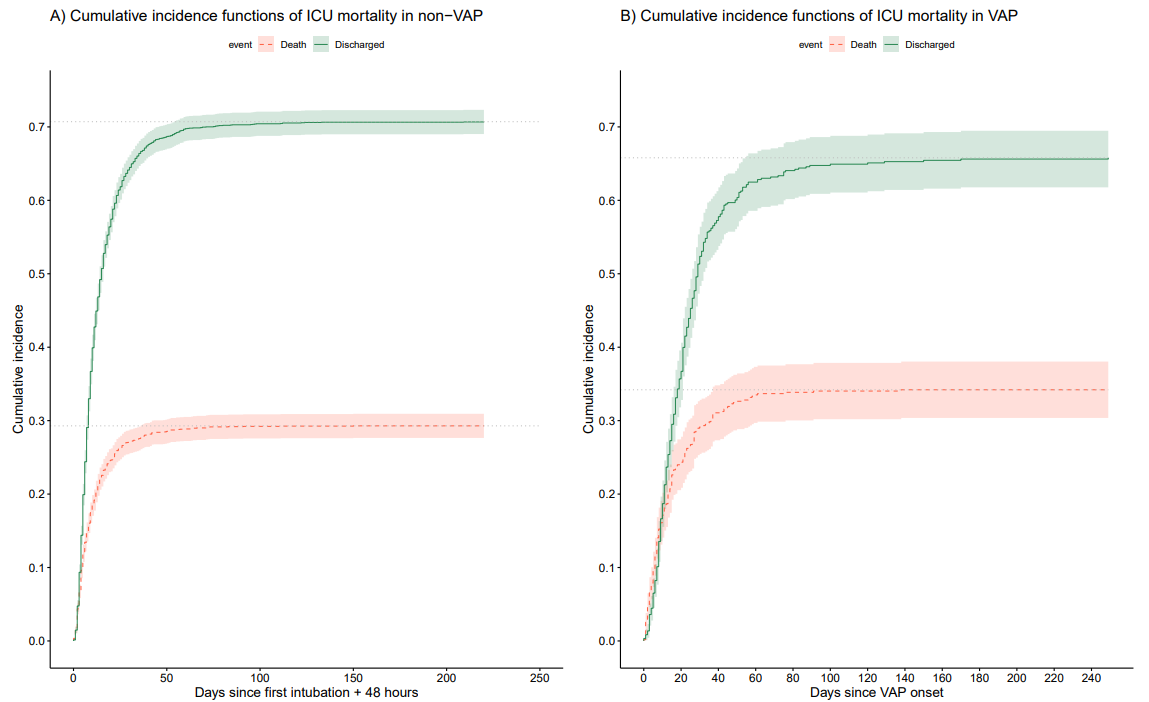
**

**Supplementary Figure 4 Invasive mechanical ventilation free days in A) VAP patients N=574 and B) VAP patients with strictly positive IMV free days N=326**


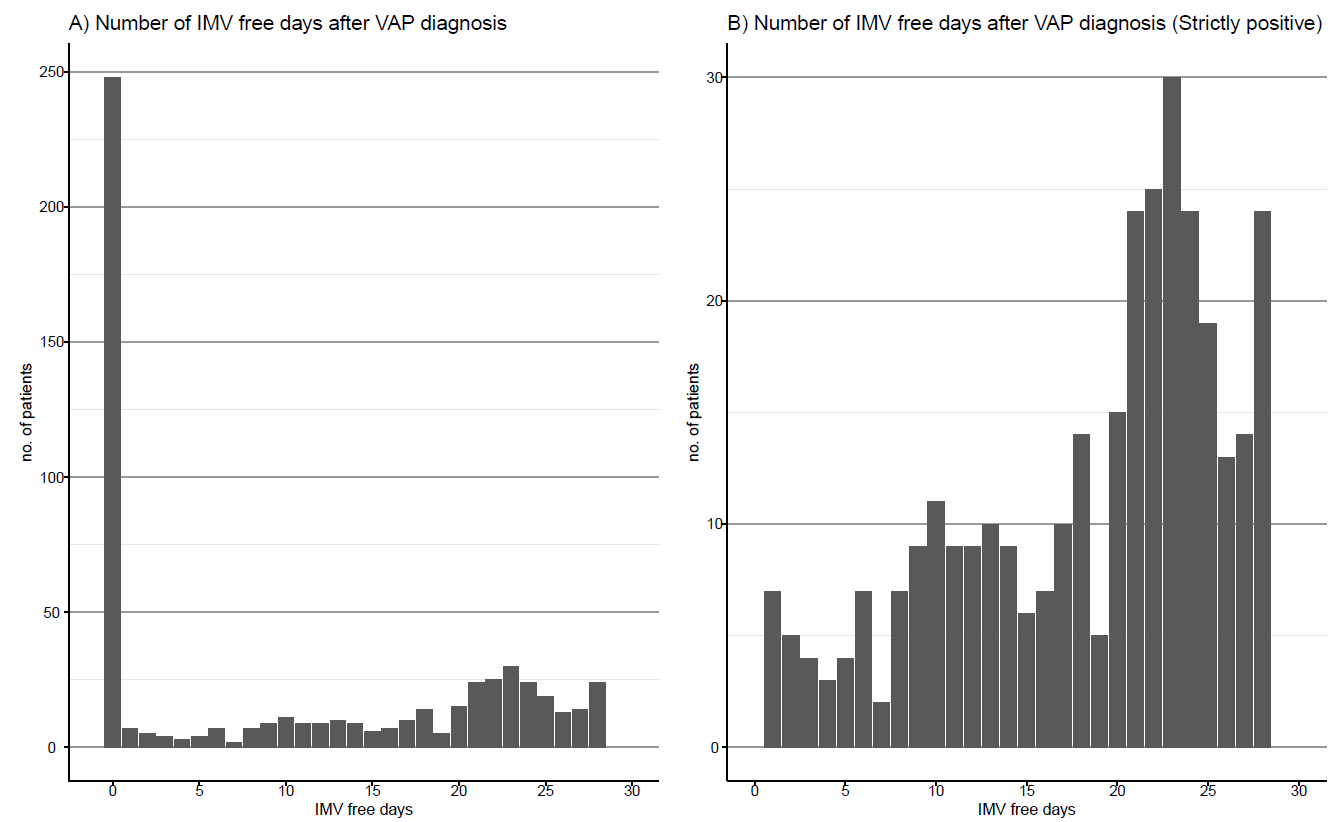


**Supplementary Table 8 Sensitivity analysis for clinical cure in VAP population, N=590**

| **Patient population** | **Clinical Cure 1 n (%)** | **Clinical Cure 2 n (%)** | **Clinical Cure 3 n (%)** |
| --- | --- | --- | --- |
| *All VAP Patients N=590* | | | |
| Cured  Not Cured  Total  Left ICU Missing | 46 (12.7)  317 (87.3)  363 (100)  115 112 | 46 (10.2)  403 (89.8)  449 (100)  47 94 | 144 (27.5)  379 (72.5)  523 (100)  0 67 |
| *All VAP patients with physician VAP diagnosis, N=505* | | | |
| Cured  Not Cured  Total  Left ICU Missing | 40 (12.7)  275 (87.3)  315 (100)  97  93 | 40 (10.3)  349 (89.7)  389 (100)  38 78 | 123 (27.3)  327 (72.7)  450 (100)  0 55 |
| *All VAP patients with physician VAP diagnosis within +/-1 day from FDA diagnosis and who started antibiotic treatment within +/-1 day from physician VAP diagnosis, N=375* | | | |
| Cured  Not Cured  Total Left ICU Missing | 31 (13.1)  205 (86.9)  236 (100)  77 59 | 31 (10.5)  263 (89.5)  294 (100) 30 48 | 102 (29.6)  243 (70.4)  345 (100) 0 27 |
| *All VAP patients with microbiological sample taken +/-2 days from FDA VAP diagnosis, N=496* | | | |
| Cured  Not Cured  Total Left ICU Missing | 41 (13.1)  273 (86.9)  314 (100)  89 93 | 41 (10.7)  341 (89.3)  382 (100)  34 80 | 122 (27.6)  320 (72.4)  442 (100)  0 54 |

**Supplementary Figure 5 A) Frequency and B) Proportion of VAP associated antibiotic use by country, across ‘s’ sites and including ‘n’ patients, in the ABT VAP population, N=375**

**
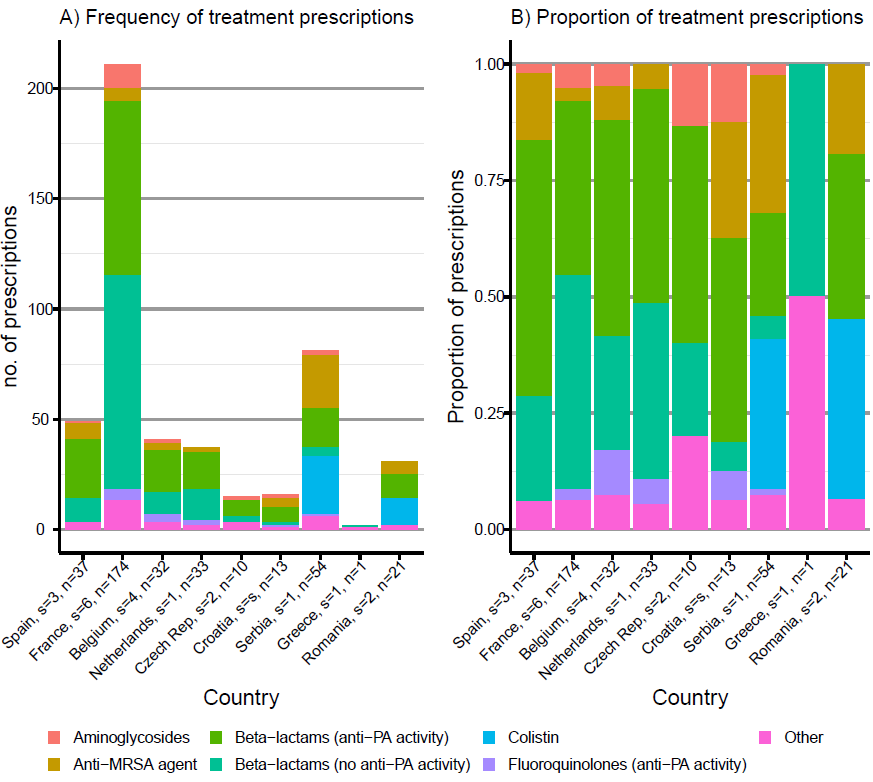
**

**Supplementary Table 9 Antibiotic treatments prescribed per patient in the VAP population who are also physician diagnosed with VAP within +/-1 day (N=436), stratified by country**

**
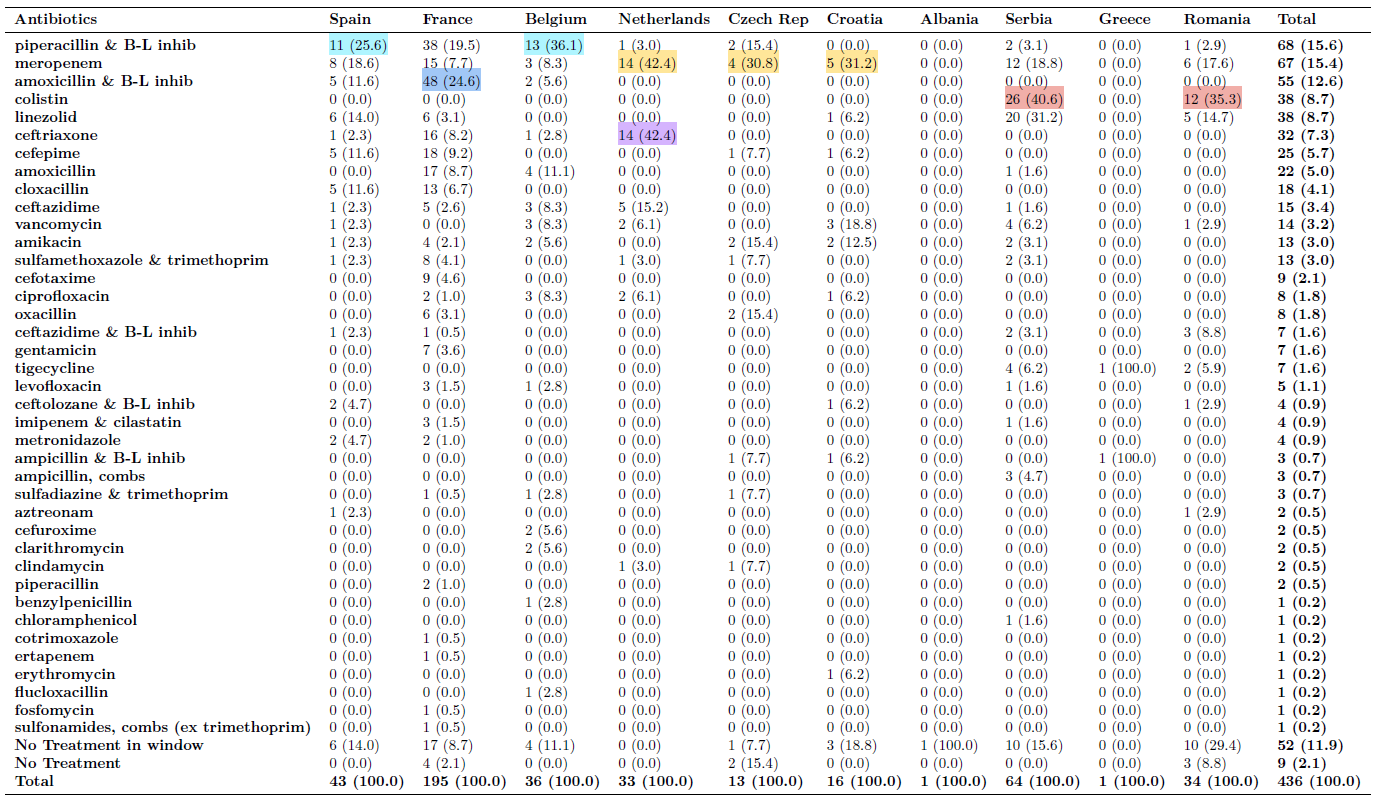
***The dominating antibiotic per country is highlighted, where each antibiotic is highlighted with a different colour

B-L inhib, beta-lactamase inhibitors
